# 

**Published:** 1884-10

**Authors:** 


					﻿OCTOBER, 1884:
OLD SERIES
---Vnl YYV-^--
1855 o ‘
\NEVk SERIES
VW-t—No. «.

Bediga«ur»
(dOURNAIi:
t r^ vvA,-^./ ^/feZMtcA—• <3b.
kQiikhJ
W.F.WESTMORELAND,
s H.V.M.MILLER,M.D.LL.D.>;
JAMES A.GRAY. M.D.^
Published By james p.harrison&cqJ
__________________Atlanta ,ga.
SUBSCRIPTION: 2.50 A YEAR.
				

